# Phenolic Compounds of *Reynoutria* sp. as Modulators of Oral Cavity Lactoperoxidase System

**DOI:** 10.3390/antiox10050676

**Published:** 2021-04-26

**Authors:** Marcin Magacz, Maria Oszajca, Izabela Nawrot-Hadzik, Ryszard Drożdż, Anna Jurczak, Jakub Hadzik, Aleksander Smakosz, Wirginia Krzyściak

**Affiliations:** 1 Department of Medical Diagnostics, Faculty of Pharmacy, Jagiellonian University Medical College, Medyczna 9, 30-688 Krakow, Poland; marcin.magacz@doctoral.uj.edu.pl (M.M.); ryszard.drozdz@uj.edu.pl (R.D.); 2 Doctoral School of Health and Medical Sciences, Jagiellonian University Medical College, 31-008 Krakow, Poland; 3 Faculty of Chemistry, Jagiellonian University, Gronostajowa 2, 30-387 Krakow, Poland; 4 Department of Pharmaceutical Biology and Biotechnology, Wroclaw Medical University, 50-367 Wroclaw, Poland; izabela.nawrot-hadzik@umed.wroc.pl (I.N.-H.); aleksander.smakosz@gmail.com (A.S.); 5 Department of Pediatric Dentistry, Institute of Dentistry, Jagiellonian University Medical College, 31-155 Krakow, Poland; anna.jurczak@uj.edu.pl; 6 Department of Dental Surgery, Wroclaw Medical University, Krakowska 26, 50-425 Wroclaw, Poland; jakub.hadzik@umed.wroc.pl

**Keywords:** lactoperoxidase, *Reynoutria*, polyphenols, dental caries, *Streptococcus mutans*, lactoperoxidase reactivators, stopped-flow spectroscopy, peroxidase

## Abstract

Lactoperoxidase (LPO) together with its (pseudo)halogenation cycle substrates, H_2_O_2_ and thiocyanate ions oxidized to hypothiocyanite ions, form one of the main systems involved in antimicrobial defense within the oral cavity. In bacterial diseases such as dental caries, lactoperoxidase is oxidized to a form known as Compound II, which is characterized by its inability to oxidize SCN^–^, resulting in a decreased generation of antimicrobial products. *Reynoutria* sp. rizome extracts, due to their high polyphenol content, have been tested as a source of compounds able to regenerate the antimicrobial activity of lactoperoxidase through converting the Compound II to the native LPO state. In the presented study, acetone extracts of *R. japonica, R. sachalinensis*, and *R. x bohemica*, together with their five fractions and four selected polyphenols dominating in the studied in extracts, were tested toward lactoperoxidase reactivating potential. For this purpose, IC50, EC50, and activation percentage were determined by Ellman’s method. Furthermore, the rate constants for the conversion of Compound I–Compound II and Compound II–native-LPO in the presence of extracts, extracts fractions, and selected polyphenols were determined. Finally, the ability to enhance the antimicrobial properties of the lactoperoxidase system was tested against *Streptococcus mutans*. We proved that *Reynoutria* sp. rhizome is the source of lactoperoxidase peroxidation cycle substrates, which can act as activators and inhibitors of the antimicrobial properties of that system. The presented study shows that the reactivation of lactoperoxidase could become a potential therapeutic target in prevention and treatment support in some infectious oral diseases.

## 1. Introduction

Lactoperoxidase (LPO) is a heme-containing enzyme that is secreted into many body-fluids, including saliva [1]. It forms the so-called lactoperoxidase system (LPO system), together with the substrates of the (pseudo)halogenation cycle, such as hydrogen peroxide, (pseudo)halide ions (mainly thiocyanate ions SCN^–^), and their oxidation products, i.e., hypo(pseudo)halides (mainly hypothiocyanite ions OSCN^–^) [1,2]. This system is an important component of the innate immune response in the area of the oral cavity and other mucous membranes [3]. Its biological activity is based on the oxidation of microorganisms protein sulfhydryl moieties, which are important to maintain the activity of various proteins and enzymes [4,5,6]. These reactive LPO products are formed in the (pseudo)halogenation cycle, in which through two-electron-processes the active center of the Fe^III^(P)_LPO_ enzyme is first oxidized by H_2_O_2_ to a highly reactive oxo-iron(IV) porphyrin π-cation radical intermediate of LPO (viz. (P^+•^)Fe^IV^(O)_LPO_ known as Compound I), which subsequently oxidizes the (pseudo)halide (SCN^–^) substrate reconstructing the native form of the enzyme [4,7]. It should be noted that Compound I, under certain conditions, can also engage in the peroxidation cycle consisting of two one-electron oxidation reactions of the organic substrate. Following the first, one-electron oxidation step, an oxo-iron(IV) porphyrin intermediate of LPO (viz. (P)Fe^IV^(O)_LPO_ known as Compound II) is formed [4,7,8]. This process may take place in vivo in the presence of phenolic organic substances, such as protein tyrosin residues, hormones [9], drugs [10], or food-derived compounds [11]. Under physiological conditions, H_2_O_2_ is continuously produced as a product of the superoxide dismutase and NADPH oxidases of the salivary glands [12,13]. Moreover, it is synthesized by numerous microorganisms, including *Streptococcus* bacteria, as a substance that regulates growth and inhibits competing microorganisms [14].

Dental caries is a disease associated with the formation of biofilms consisting of microorganisms anchored in the extracellular matrix (EPS), which provides protection against conventional antimicrobial compounds [15] and can cause chronic infections in addition to the demineralization and proteolysis of hard dental tissues [16]. Despite technological progress, the disease continues to be an unsolved health problem that affects mankind around the world [17,18]. Current methods of caries prophylaxis are based mainly on fluoride and are ineffective due to the constantly growing scale of the problem, which is associated with increasing consumption of highly processed food and simple sugars.

It should be noted, that in the course of inflammatory diseases of the oral cavity, such as dental caries or gingivitis, the synthesis of hydrogen peroxide is increased due to intensified oxidative phenomena [19]. This leads to the intensification of Compound I synthesis and imbalance between excretion of SCN^–^ ions and its oxidation by Compound I. It results in excess of Compound I relative to SCN^–^, which results in the formation of the stable and relatively weakly reactive form viz. Compound II and its accumulation. This process impairs the operation of the LPO system by preventing the regeneration of the native form of enzyme and in consequence also of Compound I, responsible for the synthesis of antimicrobial products of the (pseudo)halogenation cycle [20].

In recent years, intensive research has been carried out on phenolic compounds—derived from plants and the processing products of plants, such as wine, chocolate or tea—due to the high biological activity of these compounds exhibiting anti-inflammatory, anticancer, antimicrobial, and antioxidant properties [21,22]. Since phenolic compounds are good substrates of the peroxidation cycle, interaction with participating enzymes (including LPO) may be a molecular target in the treatment of diseases, in which these enzymes are involved in the pathogenesis mechanism or the therapeutic action. To date, numerous phenolic LPO activators of plant origin [1,19,20,23,24,25] capable of reducing Compound II to native LPO, thus restoring its potential ability to synthesize antimicrobial product, have been indicated. Nevertheless, to our knowledge, such studies have been limited to enzyme kinetics only, and microbial studies have not been published yet.

*Reynoutria japonica* (also known as Japanese Knotweed), *Reynoutria sachalinensis*, and a hybrid of these two species, *Reynoutria x bohemica*, may be potential sources of the LPO activators. These plants are highly expansive and invasive in North America and Europe and originate from Asia [26]. The first of these species has been used in East Asia at least since the Han Dynasty [27] as a medicinal plant, inter alia, in the treatment and prevention of oral diseases [28]. Currently, plants of this species are of great interest as a source of biologically active compounds due to the high content of polyphenol antioxidants in the rhizome [26,27,29,30]. Based on our previous research that assessed the composition of rhizome extracts from three species of *Reynoutria* [29], we hypothesized that the phenolic compounds contained in these extracts have the ability to regenerate the LPO system through reduction of the inactive Compound II, which ultimately leads to an increase in OSCN^–^ ions formation rate.

The aim of this study was to test the potential of acetone extracts from the rhizomes of *R. japonica*, *R. sachalinensis*, *R. x bohemica*, and its individual fractions (dichloromethane, ethyl acetate, diethyl ether, butanol, and water) to regenerate the LPO system. Moreover, the ability of (-)-epicatechin, resveratrol, emodin, and vanicoside B—representing the four most important groups of polyphenols present in the tested extracts (flavan-3-ols, stilbene derivatives, anthraquinones, and phenylpropanoyl sucrose derivatives, respectively)—to reactivate the enzyme was tested. Our research included both kinetic analyses and the assessment of the actual biological effect against *Streptococcus mutans*, considered to be one of the main etiological factors of dental caries [31].

## 2. Materials and Methods

### 2.1. Reagents

Lactoperoxidase from bovine milk was purchased as a lyophilisate (Sigma-Aldrich, Darmstadt, Germany). Immediately before use, the enzyme was dissolved in a phosphate buffer at pH 6.00 at a concentration of 100 mM or 20 mM (in the case of the stopped-flow technique). Molar concentration of the obtained enzyme solution was assessed spectrophotometrically at a wavelength of λ_max_ = 412 nm using the molar extinction coefficient ε = 112,000 M^−1^·cm^−1^ [32].

Hydrogen peroxide solution was prepared fresh each day using 30% stock solution. The concentration of the solution was determined spectrophotometrically at λ_max_ = 230 nm (ε = 74 M^−1^·cm^−1^) [24].

Ellman’s reagent (DTNB, 5,5′-dithiobis-(2-nitrobenzoic acid)) used to measure the (pseudo)halogenation activity of LPO on thiocyanates was obtained by reduction of TNB (2-nitro-5-thiobenzoic acid) (Sigma-Aldrich, Germany) with sodium borohydride (Sigma-Aldrich, Germany) in a 50 mM phosphate buffer at pH 8 [24]. The DTNB was stored at 4 °C for a maximum of 7 days.

### 2.2. Preparation of Extracts

Extracts and their fractions were obtained earlier according to the procedure described in Nawrot-Hadzik et al. [29]. Briefly, rhizomes of *R. japonica*, *R. sachalinensis*, and *R. x bohemica* from a wild population in urban areas of Wroclaw (Poland) were harvested and confirmed by Botanical Garden of Medicinal Plants Herbarium staff. Rhizomes were air-dried, next powdered and extracted with use of 70% aqueous acetone. Dry extracts after suspending in water were afterward fractionated with dichloromethane (DCM), diethyl ether (Et_2_O), ethyl acetate (EtAc), and, finally, butanol. Detailed phytoanalysis and antioxidant activity of these fractions were presented by Nawrot-Hadzik et al. [29]. In the earlier study, ref [27] qualitative and quantitative analysis of all acetone extracts were carried out using reversed-phase high performance liquid chromatography method with diode array detector and time-of-flight mass spectrometry. Six compounds from three phytochemical groups were quantified using the validated method, i.e., piceid and resveratrol from stilbenes, emodin and physcion from anthraquinones, and vanicosides A and B from hydroxycinnamic sucrose esters. Moreover, detailed phytochemical composition of all fractions and the relative content of compounds were presented in another study [29]. As previous analysis showed that most of the compounds belong to one of the four groups of phytochemicals, i.e., stilbenes, anthraquinones, hydroxycinnamic sucrose esters, and flavan-3-ols, we chose one compound from each group (resveratrol, emodin, vanicoside B, (-)-epicatechin) occurring in significant amounts in the extracts.

Polyphenols, i.e., resveratrol, (-)-epicatechin, and emodin, were obtained from Carl Roth, Germany. Vanicoside B was isolated from the rhizomes of *R. sachalinensis* and identified according to the procedure described in a published paper [27]. Stock solutions of polyphenols were obtained by dissolving the samples in 99.7% DMSO (POCH, Avantor Performance Materials Poland S.A, Gliwice, Poland) to obtain solutions of a concentration between 3–7 mM. Similarly, solutions of dried extracts and fractions were obtained by dissolving them in DMSO to obtain stock solutions of a concentration between 2–5 mg/mL.

### 2.3. Assessment of the Effect of Extracts and Selected Polyphenols on the Production of OSCN^–^ by LPO

The method with the Ellman’s reagent was used to test the (pseudo)halogenation activity of LPO with respect to thiocyanates. The principle of the method is based on following the decrease in absorbance from DTNB associated with its oxidation to TNB by OSCN^–^ resulting from the oxidation of SCN^–^ in the LPO (pseudo)halogenation cycle [24,33].

Measurements were carried out in 96-well plates using a ThermoFisher Multiskan Sky reader (Thermo Fisher Scientific, Waltham, USA). Each sample was tested at minimum four times. In order to assess the effect of extracts/polyphenols on the (pseudo)halogenation activity of LPO, solutions of the test substances were added to the wells in a series of 2-fold dilutions. After a 2 min pre-incubation time with the enzyme and Ellman’s reagent, the reaction was started by adding the H_2_O_2_ solution. In order to determine the effect dependent on the enzymatic reaction, control samples without the enzyme addition were analyzed in parallel. The formation of OSCN^–^ was kinetically monitored at 37 °C for 5 min by measuring the decrease in DTNB absorbance (ε = 14,000 M^−1^·cm^−1^) at 412 nm. The dependence of absorbance at 412 nm on time was used to create linear model, and the initial reaction rate was calculated from the slope of the DTNB concentration versus time curve using a linear fit and was expressed in µM/s. For further analysis, only measurements with *r* coefficient greater than 0.95 were used.

The final composition of the reaction mixtures contained 3 mM SCN^–^, 100 μM H_2_O_2_, 0.5 nM LPO, and various concentrations of extracts/polyphenols in 100 mM phosphate buffer at pH = 6.0.

For the analyzed extracts and compounds, the dependence of the rate of OSCN^–^ ion synthesis by LPO on the concentration of the tested substance was assessed. For extracts and compounds showing an activating effect, the EC_50_ value (half maximal effective concentration) and the activation percentage were determined, while for those showing an inhibitory effect, the IC_50_ (half maximal inhibitory concentration) was determined.

### 2.4. Analysis of Kinetics via Stopped-Flow Method 

Stopped-flow studies were carried out with the use of Applied Photophysics SX20 stopped-flow spectrophotometer equipped with a sequential mixing mode, supplied with an Xe lamp and a PMT (Photomultiplier Tube) or diode array detector. Measurements were performed at 15 ± 0.1 °C in 20 mM phosphate buffer. The kinetics of the reaction of lactoperoxidase with selected substrates was studied under pseudo-first-order conditions. In a typical experiment, equal volumes of LPO (final concentration 1 μM) and hydrogen peroxide (final concentration 1 μM) were rapidly mixed in the first mixing drive. After 200 ms of incubation required to generate Compound I, the aged solution was mixed with a substrate (at least 7 times molar excess) in the second mixing drive, and the formation of Compound II was followed spectrophotometrically at 422 nm, which corresponds to the isosbestic point for conversion of Compound II to native LPO form. Such approach allowed registration of single exponential kinetic traces assigned only to the studied step and elimination of the disturbance coming from the relatively fast subsequent reduction of Compound II to Fe^III^(P)_LPO_.

Reduction of Compound II to native LPO was followed in a similar way, but the final concentration of hydrogen peroxide was reduced to 0.75 μM, and incubation time in the first mixing drive was set to 5 s in order to generate Compound II, while the wavelength was set to 413 or 430 nm.

Each measurement was performed at least four times. Depending on registered kinetic trace (time dependence of absorbance), character/course mono- or bi-exponential functions were applied to determine the observed rate constants (k_obs_). Fitting to selected function was done using the Pro-Data Software (Applied Photophysics, Leatherhead, UK).

Second-order rate constants were calculated using a linear fit (least squares method) to k_obs_ vs. concentration with the Origin software (OriginLab Corporation, Northampton, Massachusetts, USA).

### 2.5. Assessment of the Effect of Extracts on the Inhibition of S. mutans Growth by LPO

The extracts that showed the ability to increase the production rate of OSCN^–^ in kinetic models were assessed for the ability to increase the antimicrobial effect of the LPO system in a microbiological model.

The determinations were made with the reference *S. mutans* ATCC 2517 strain. The strains were used not later than from the fifth passage. The microorganisms were grown at 37 °C in an atmosphere enriched with 10% CO_2_. After isolation from Columbia agar with 5% sheep blood, colonies were inoculated into brain–heart infusion (BHI) liquid and incubated overnight for bacterial growth. Immediately before use, the microorganisms in the liquid culture were centrifuged three times and washed with sterile PBS. Growth kinetics of *S. mutans* in BHI medium was assessed spectrophotometrically at 600 nm in the presence of several dilutions of the tested extracts and the SCN^–^-H_2_O_2_-LPO system. Freshly prepared suspension of the tested extract solution (a series of 2-fold dilutions in the concentration range that did not directly inhibit microbial growth), KSCN solution (final concentration 2 mM), and LPO solution (final concentration 50 nM) were added to the bottom of the 96-well plate. *S. mutans* in BHI (final density 0.01 in McFarland scale (McF)) was added and then the reaction was initiated by the addition of H_2_O_2_ solution (final concentration 300 µM). Microbial growth curves were then recorded for 24 h at 5 min intervals while the plate was incubated at 37 °C. Each determination was performed in quadruplicate.

The time taken for the microorganisms to reach half of the log phase of growth was determined for each recorded growth curve. Evaluation of the activating or inhibiting effect of the LPO system was assessed by calculating the difference in time to reach half-log phase growth between the system containing the complete LPO system and the test compound/extract, and the system containing only LPO system (without the tested compound/extract) (Δ*t*). This parameter obtained for the series of dilution of tested extract/fraction/compound was plotted against concentration, which allowed the fitting of data using the Hill model in order to calculate EC_10/50/90_ and IC_10/50/90_.

### 2.6. Determination of the MIC and MBC

The minimum inhibitory concentration (MIC) of the extracts was determined by serial duplicate dilution in a sterile 96-well flat-bottom plates. Extracts were diluted in series with sterile 0.9% NaCl solution and then 100 µL of each was added to the bottom of the plate. Subsequently, 100 µL of a freshly prepared suspension of *S. mutans* in twice concentrated MHB was introduced into the wells. The final bacterial density was 5 × 10^5^ CFU/mL (colony forming unit). Plates were incubated for 24 h at 37 °C in a 10% CO_2_ enriched atmosphere. After this time, the absorbance was measured at 600 nm. An absorbance correction was applied to eliminate the effect of the extracts’ intrinsic absorbance by subtracting the absorbance values after incubation from those before incubation.

The minimum bactericidal concentration (MBC) of the extracts was determined by transferring 10 μL of the contents of wells with an extract concentration equal to or above the MIC to a solid medium (Columbia agar with 5% sheep blood). Readings were made after 48 h of incubation; MBC was defined as the lowest concentration of extract with which no *S. mutans* growth was observed on solid media after 48 h of incubation.

### 2.7. Statistical Analysis

Statistical analysis was performed using the R environment v.4.0.3 (R Development Core Team). All statistical tests were done at 0.05 significance level.

Linear fit to obtain OSCN^–^ initial formation rate (slope) was done using the built-in linear model utilizing the least squares method.

Dose-response curves of both kinetics and microbiological models were fitted using the Hill method with ‘drc’ package (Christian Ritz bioassay.dk) (LL.4 function–four parameter log-logistic function), which allowed the determination of IC_10/50/90_/EC_10/50/90_ as well as the percentage of activation. The significance of dose–response dependence was determined by the analysis of statistical difference from zero of all four function parameters.

The variable distribution normality was verified using Kolmogorov–Smirnov test. The differences of the means between extracts/fractions/compounds were analyzed using ANOVA test with post-hoc Tukey test.

## 3. Results and Discussion

### 3.1. Selection of Conditions for Determining the Effect of Extracts on the Formation of OSCN^–^ in the LPO Halogenation Cycle

**Figure 1:**
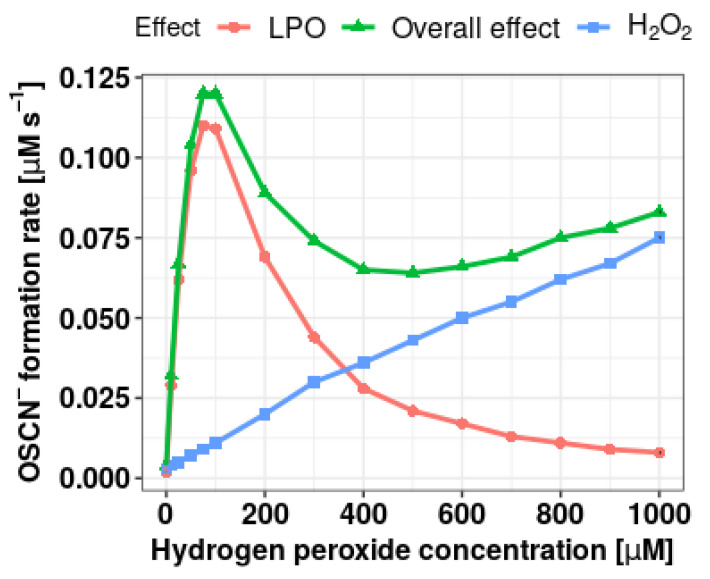
Relationship between the initial rate of OSCN^–^ formation and the concentration of hydrogen peroxide. The green triangles indicate the OSCN^–^ formation initial reaction rate, derived from the DTNB oxidation in the presence of just hydrogen peroxide (without the presence of LPO). The blue squares indicate the sum of the H_2_O_2_ effect and the enzymatic reaction. The enzyme activity-related reaction rate was calculated by subtracting the H_2_O_2_-dependent rate from the sum rate value and was marked with a red circle.

Figure 1 shows the dependence of the initial rate of SCN^–^ oxidation to OSCN^–^ ions on the concentration of hydrogen peroxide. The spectrophotometrically recorded overall effect is the sum of the oxidation reactions of thiocyanates in direct reaction with hydrogen peroxide, direct oxidation of DTNB by H_2_O_2_, and oxidation in the (pseudo)halogenation cycle of LPO. In order to estimate the effect dependent only on the enzymatic reaction, the initial reaction rate presented in further studies is the difference between the total initial reaction rate (full LPO system) and the initial rate for reaction conducted without LPO addition. The maximum reaction rate was observed at a concentration of 100 µM H_2_O_2_. Above this value a systematic decrease in the LPO-dependent OSCN^–^ formation rate was observed. Although the physiological H_2_O_2_ concentration in the oral cavity in situ is in the range of 8–14 µM [4], its concentration at the level of 100 µM was selected for further studies due to the faster course of the reaction. More importantly, this concentration simulates inflammatory conditions characterized by overproduction of hydrogen peroxide and the consequent formation of non-reactive LPO forms. Contrary to other groups of researchers [24,25,34] dealing with the subject of LPO system reactivation, in our study we decided to lower the pH from 7.4 to 6.0 in order to simulate the pro-cariogenic conditions prevailing in the oral cavity after a meal, when due to the production of organic acids by microorganisms, the pH of the oral cavity decreases.

### 3.2. Effect of Individual Extracts and Polyphenols on the Formation of OSCN^–^ in the LPO Halogenation Cycle

**Figure 2:**
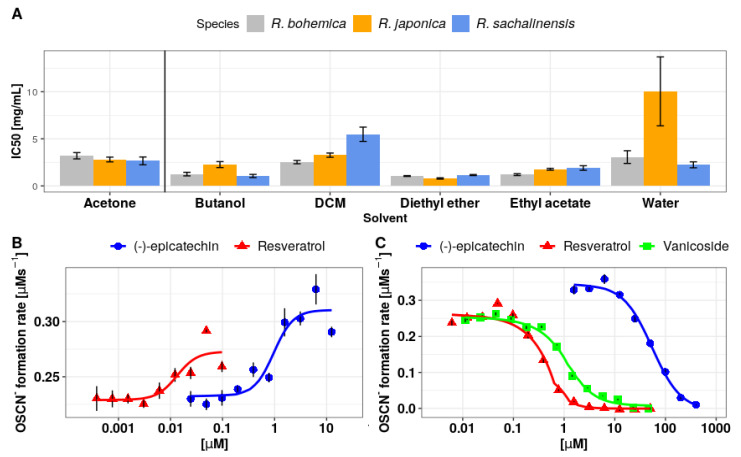
(**A**): IC_50_ of acetone extracts from the rhizome of *Reynoutria* sp. and individual fractions obtained with various solvents. Black lines indicate the standard error of the quadruple marking of each fraction. (**B**): Dose–response curves (the Hill model) showing the activating effect of (-)-epicatechin and resveratrol in relation to the pseudo(halogenation) activity of LPO. (**C**): Dose–response curves (the Hill model) showing the inhibitory effect of (-)-epicatechin, resveratrol, and vanicoside B on the pseudo(halogenation) activity of LPO.

The IC_50_, EC_50_, and activation (%) values for the studied acetone extracts, fractions, and analyzed compounds are provided in Table 1. Figure 2A shows IC_50_ for all tested extracts and fractions, whereas Figure 2 shows dose–response curves fitted using the Hill method for tested polyphenols in activation concentration range (B) and inhibition concentration range (C).

**Figure 3:**
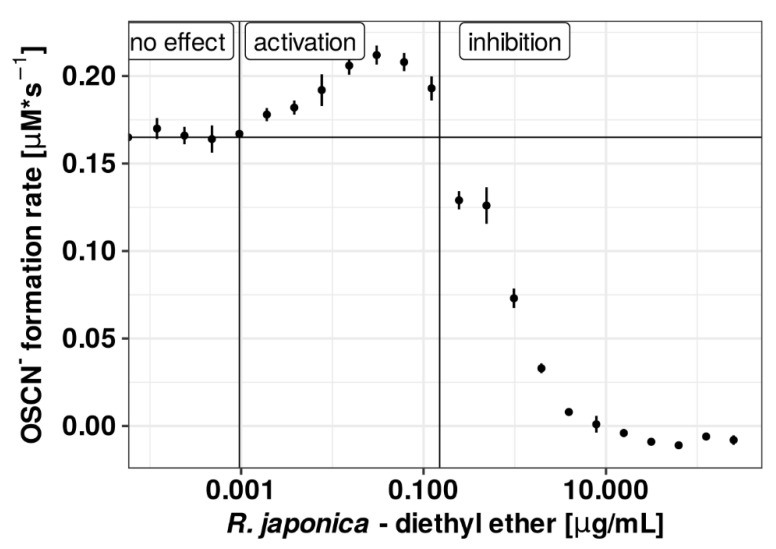
The dependence curve of observed OSCN^–^ formation rate in the presence of successive dilutions of the studied extract showing both inhibitory and activating effects (extract of diethyl ether fraction from *R. japonica*).

Under the applied conditions, all primary acetone extracts displayed only inhibitory properties toward (pseudo)halogenation activity of LPO. Comparison of the IC_50_ values determined for the primary extract indicates the strongest inhibitory properties (the lowest IC_50_) of the one obtained from *R. sachalinensis*, while the weakest (the highest IC_50_) was of *R. x bohemica* (Table 1 and Figure 2A). However, for the tested fractions (DCM, ethyl acetate, diethyl ether, butanol, and water residue), apart from the inhibitory effect, also an activating effect was observed. The regenerative properties were exhibited by the diethyl ether fractions of all three *Reynoutria* species and the DCM *R. x bohemica* fraction. It should be noted that the type of the observed effect depended on the concentration of the tested fraction, with activation occurring at lower concentrations and inhibition at higher concentrations. An example of the dependence of the initial reaction rate of OSCN^–^ formation on the concentration of the fraction exhibiting both activation and inhibitory properties is presented in Figure 3.

The activating effect of the diethyl ether fraction can be related to the high total content of polyphenols (TPC assay, total polyphenol content using Folin–Ciocalteau method) and tannins (expressed as GAE, gallic acid equivalent) shown in previous studies [29]. This fraction, compared with the others, contains particularly high concentrations of compounds such as flavanols, which include (-)-epicatechin, and in the case of *R. japonica* and *R. sachalinensis*, stilbene derivatives, which include resveratrol. Both mentioned compounds were able to increase the initial OSCN^–^ formation rate by LPO in the assay using Ellman’s reagent (Table 1). Interestingly, the highest activation percent was found in the case of diethyl ether fraction of *R. japonica*, which was characterized by the highest ability to synthesize stilbene derivatives, and the lowest activation percent value was in the case of diethyl ether fraction of *R. sachalinensis*, in which no stilbene derivatives were found [29].

DCM fractions exhibited relatively high IC_50_ values compared with other fractions. Composition analysis revealed that DCM fraction is rich in lignin oligomers and anthraquinines (emodin). Studies on the catalytic activity of emodin revealed neither an activating nor an inhibitory effect, which may indicate that inhibition properties of that fraction is dependent mainly on another identified group of compounds, i.e., lignin oligomers. As demonstrated by Gau et al. [19], hydrolysable molecules of tannins are sufficiently large to not be bound effectively in the binding site of LPO, and therefore they do not regenerate the (pseudo)halogenation activity of LPO [19]. Nevertheless, the DCM fraction of the *R. x bohemica* extract showed the ability to increase the (pseudo)halogenation activity of LPO in our research. This may be related to the presence of trace amounts of low-molecular-weight tannin hydrolysis products identified by Gau et al., which are known as good substrates for the high-valent oxo-iron(IV) reactive intermediates of LPO [19].

The ethyl acetate fraction, despite having the highest content of polyphenols (TPC assay) among all fractions, as described in previous studies [29], did not show any activating properties. Moreover, this fraction exhibited strong potential to inhibit OSCN^–^ synthesis. This may be due to the fact that the total content of polyphenols in this fraction consists mainly of high-molecular tannins, including oligomers of proanthocyanidins. The studies of Gau et al. showed that the potential of proanthocyanidins to regenerate LPO depends on both the molecular weight of the compound, the number of subunits, and the number and types of interflavanyl bonds [19]. Despite the observed increase in the catalytic constant (k_cat_) for the oxidation reaction, along with the increasing number of 2β-O-7 bonds by increasing the amount of flavanyl units, the authors observed a decrease in the ability to bind a specific polyphenol molecule to the substrate binding site of LPO [19].

The butanol fraction, similar to the ethyl acetate fraction, was characterized by a high content of proanthocyanidins; however, the level of polymerisation of these compounds in the discussed fraction was higher (up to decamers) [29]. In this case observed inhibition of OSCN^–^ formation could be the result of the ability of highly polymerized proanthocyanidins to bind proteins in a nonspecific manner. As demonstrated by Kilmister et al. [35], this occurrence increases with the increase of the molecular mass of proanthocyanidin oligomers, which in turn causes protein–tannin–protein crosslinking leading to the precipitation of the complex and loss of biological activity. The occurrence of the process has been proven for globular proteins such as bovine albumin [35], lysozyme, or α-lactalbumin [36]; however, due to the nonspecific mechanism, it can be assumed that it may also be applied to other globular proteins, including LPO, leading to the inhibition of (pseudo)halogenation activity.

The water residue fraction as we have shown in previous studies [29] consisted mainly of carbohydrates. The inhibition properties of that fraction, although relatively low, could be associated with trace amounts of phenolic compounds from hydrolysis of phenolic–carbohydrate conjugates.

In order to shed more light on the observed activating and inhibitory properties of the studied extracts and their fractions, four polyphenols representing the most important chemical groups of compounds identified in the extracts were selected based on the analysis of the extract composition using HPLC-MS: resveratrol (stilbenes), emodin (anthraquinones), (-)-epicatechin (flavan-3-ols), and vanicoside B (phenylpropanoyl sucrose derivative). Their effect on LPO-dependent OSCN^–^ synthesis was determined (Table 1).

(-)-Epicatechin, resveratrol, and vanicoside B exhibited the ability to inhibit the (pseudo)halogenation activity of LPO (which is presented in Figure 2C as OSCN^–^ initial formation rate decreases with increasing compounds concentration). Importantly, epicatechin and resveratrol, similar to the previously discussed 4 fractions, in a certain concentration range also showed an activating effect (which is presented in Figure 2B as increasing OSCN^–^ initial formation rate with increasing compounds concentration). For fractions/compounds showing activating effect, the activation force was also assessed and is expressed as a percentage of activation. It corresponds to the maximum percentage increase in the observed OSCN^–^ formation rate in terms of activation occurrence in relation to the OSCN^–^ formation rate without the addition of the extract/compound (see Table 1).

(-)-Epicatechin showed the strongest activation (46.31%) properties among the analyzed extracts and compounds. Similar activatory properties have been identified by Gau et al. [24]. Among the studied flavonoids, including flavan-3-ol, the strongest activatory properties were reported for 3′,4′-dihydroxylated compounds such as (-)-epicatechin and (+)-catechin [24]. On the other hand, the authors pointed out that flavanols deprived of the C4′hydroxyl group, due to the inverse arrangement in the LPO binding site, show an inhibitory effect on the enzyme [24]. No such derivatives were identified in the examined extracts.

Resveratrol was the second polyphenol exhibiting properties to promote OSCN^–^ synthesis. The measured OSCN^–^ synthesis activation percentage was lower than in case of (-)-epicatechin; however, EC_50_ was above 2 orders of magnitude higher than in the case of (-)-epicatechin, which indicates stronger affinity of resveratrol among both analyzed compounds.

### 3.3. Analysis of the Kinetics of the Reactions of Extracts, Fractions and Selected Polyphenols with Compound I and Compound II

Compound II is stable and inactive in terms of its ability to synthesize antimicrobial OSCN^–^. Thus, its accumulation deprives the LPO system of its antimicrobial properties by creating conditions for the deficiency of the native LPO form, capable of entering the (pseduo)halogenation cycle and generating the highly reactive Compound I. Therefore, the ability of studied primary extracts, fractions, and selected polyphenols to reduce Compound II to native (P)Fe^III^_LPO_ form was tested. Since the potency of polyphenols to regenerate LPO-mediated OSCN^–^ formation depends not only on the effective reaction with Compound II but also on the competitive reaction with Compound I, efficient reaction with Compound I may contribute to the inhibitory properties resulting from the competition of polyphenol oxidation with the oxidation of SCN^–^ by Compound I. Therefore, in the presented study, the kinetics of both the reduction of Compound I to Compound II and Compound II to native LPO with the use of the selected extracts and polyphenols was assessed with the application of stopped-flow technique.

**Figure 4:**
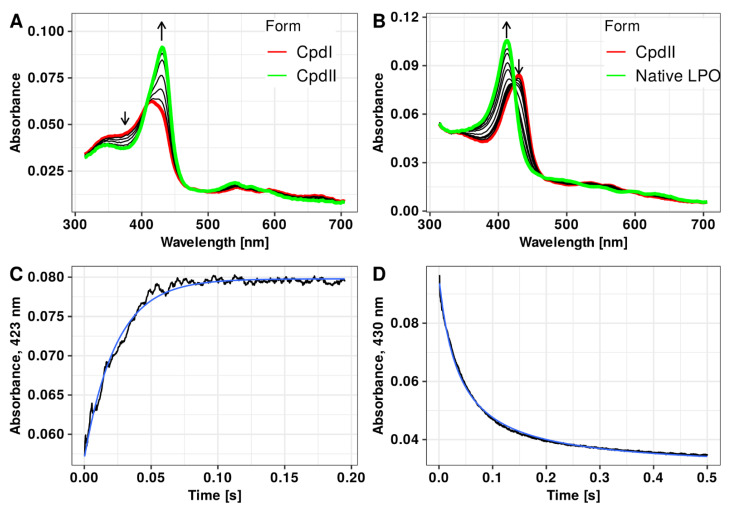
(**A**): Spectral changes accompanying the spontaneous generation of Compound II from Compound I. First spectrum (red) was obtained 200 ms after mixing LPO with hydrogen peroxide and corresponds to Compound I. Last spectrum (green) was registered after 5 s of incubation; complete generation of Compound II can be observed. (**B**): After addition of organic substrates, rapid reduction of Compound II (red) to native LPO (green) is observed. Typical kinetic trace reflecting the reaction between (-)-epicatechin and Compound I or Compound II with exponential fit (blue line) is shown at (**C**,**D**), respectively. Experimental conditions: 15°C; (**A**,**C**) LPO 1 µM, H_2_O_2_ 1 µM; (**B**,**D**) LPO 1 µM, H_2_O_2_ 0.75 µM, (-)-epicatechin 7 µM.

Native LPO (Fe^III^(P)_LPO_) was identified by a spectral maximum at 412 nm. After mixing the enzyme with hydrogen peroxide, rapid generation of Compound I (within 200 ms at 15 °C) was observed, characterized by the Soret band collapse and its maximum shift to 414 nm as well as a characteristic absorbance increase above 600 nm. Subsequently, in the absence of organic substrates, slow reduction of Compound I to Compound II ((P)Fe^IV^ = O(LPO)) (Soret band maximum at 430 nm) was observed (Figure 4A). In order to follow a reduction reaction of Compound I to Compound II, equimolar concentrations of enzyme and hydrogen peroxide were used, whereas to follow a reduction reaction of Compound II to native LPO, the enzyme/H_2_O_2_ concentration ratio was reduced to 1:0.75 to avoid a catalytic cycle initiation by unconsumed oxidant. Thus, the application of appropriate conditions (relatively low temperature, sub-equivalent or equivalent amount of oxidant to LPO, and adequate delay time) allowed the reduction kinetic of Compound I and Compound II to follow independently.

Observed reaction rate constant (k_obs_) for the reduction of Compound I to Compound II as well as for the transition of Compound II to native LPO in the presence of extracts and selected polyphenols were determined by fitting single- or double-exponential functions (depending on kinetic trace character) to the recorded kinetic traces at 422 or 430 nm, respectively (Figure 4B,D).

In the case of extracts fractions, k_obs_ values for both the reduction of Compound I to Compound II as well as Compound II to native LPO were determined merely at one concentration point (5 μg/mL), which was only sufficient for a relative comparison of the fraction reactivity (Table 2). It should be noted that in the case of vanicoside B and some extracts, the obtained kinetic curves exhibited the double-exponential shape, which indicated the occurrence of two subsequent or simultaneous reactions. In this case, the k_obs_ values were determined for both processes. Results presented in the Table 2 show that the highest k_obs_ values were obtained in the case of ether fractions, whereas the lowest values were exhibited in the presence of water residue fraction for both studied processes.

**Figure 5:**
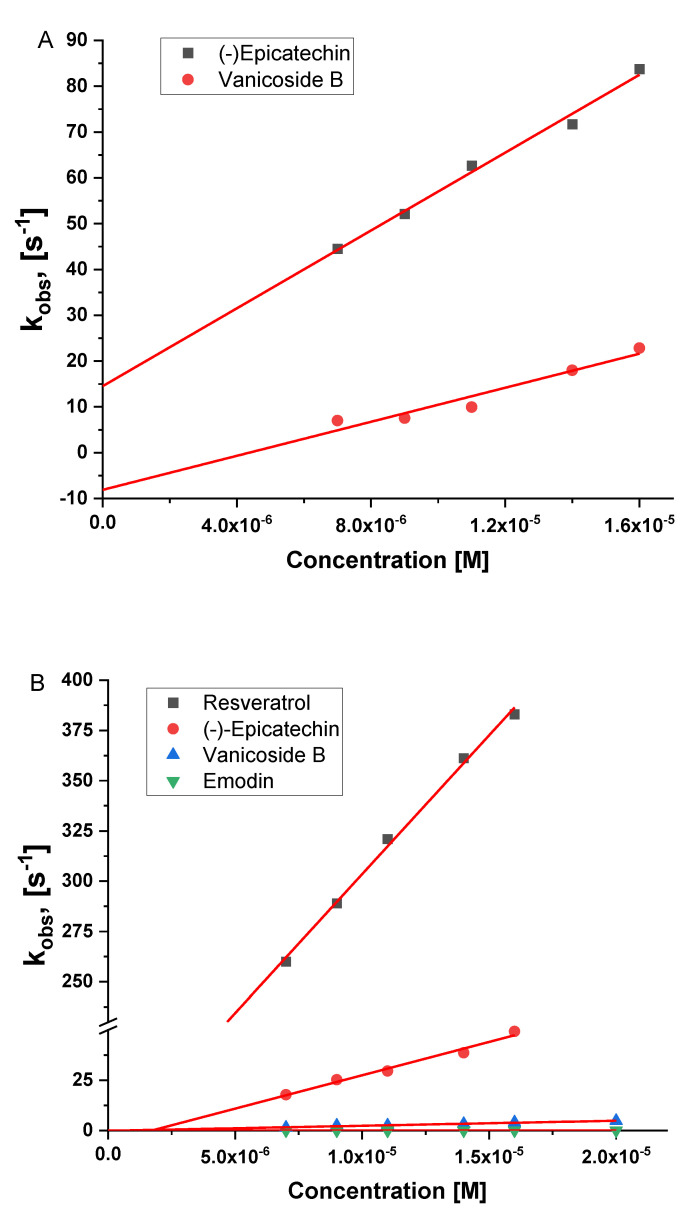
Plots of k_obs_ vs. concentration of selected polyphenols for formation (**A**) and decay (**B**) of Compound II of LPO. Experimental conditions: 0.02 M phosphate buffer pH = 6, 15°C; (**A**) (LPO) = 1 µM, (H_2_O_2_) = 1 µM; (**B**) (LPO) = 1 µM, (H_2_O_2_) = 0.75 µM.

In the case of primary acetone extracts and selected polyphenols, k_obs_ values were determined in a function of concentration. The determined k_obs_ values (average values of at least 6 measurements) plotted against the extracts or polyphenol concentration showed a linear dependence (Figure 5). Linear fit of the obtained data allowed determination of the second-order rate constants (Table 3).

Observations described in Section 3.2 are reflected in the kinetic results obtained with the stopped-flow technique. For all three species, the highest values of the observed rate constant for both tested reactions of the peroxidation cycle were found for the diethyl ether fraction. This proves that the high content of compounds present in this fraction are good substrates for LPO Compound I and Compound II. It may be related to the high content of resveratrol and (-)-epicatechin, which as presented in Section 3.2 were characterized by the ability to increase the OSCN^–^ initial reaction rate. These results correspond to relatively high second-order rate constants for the reactions of those compounds with LPO Compound II.

Resveratrol emerged as the most efficient Compound I and Compound II reducing agent among the tested polyphenols (Table 3). Moreover, resveratrol was identified as a compound with a high affinity to LPO. Koksal et al. [34] compared the ability of five peroxidation cycle substrates, including resveratrol, to inhibit the oxidation of another peroxidation cycle substrate—ABTS (2,2′-azino-bis-3-ethylbenzothiazoline-6-sulfonic acid). In these studies, resveratrol was classified as a competitive inhibitor with a low value of the inhibition constant compared with other tested polyphenolic compounds. It means that in the case of the availability of resveratrol and an another compound (for example, less reactive) that binds to LPO [24], resveratrol oxidation reaction leading to the reconstitution of the native LPO will be favored. It can be concluded that in the presence of the extract containing numerous other polyphenols, resveratrol will be the most likely component responsible for the extract-activating properties.

The lowest observed rate constants for reduction of Compound I and Compound II among tested species were determined in the presence of *R. sachalinensis* extract and fractions. This observation, apart from the aforementioned lack of stilbene derivatives, may also be related to the high content of vanicoside B in this extract when compared with the others [29]. To our knowledge, results of the research on vanicoside B and other phenylpropanoyl sucrose derivatives acting as substrates for LPO and other peroxidases have not been published so far. Our research revealed that vanicoside B can act as a substrate for both Compound I (the second-order rate constant not determined due to the non-exponential reaction course) (Table 3) and Compound II (second-order rate constant of 3.6 ± 0.5 × 10^3^ mL µg^−1^ s^−1^). The aforementioned non-exponential reaction trace may be related to the formation of products that are also further substrates for LPO due to the fact that vanicoside B has 4 phenylopropanoyl groups, and each may be a potential target of enzymatic oxidation. The lack of activation of OSCN^–^ ions formation by vanicoside B, demonstrated in the assay using Ellman’s reagent, may be related to the relatively low reduction rate of Compound II to native LPO compared with resveratrol and (-)-epicatechin.

Despite the lack of activating or inhibitory properties of emodin in kinetic studies with Ellman’s reagent, stopped-flow experiments showed that this compound may be a substrate for LPO Compound I and Compound II, in contrast to the non-reactive doxorubicin, which also belongs to anthraquinones [37]. Nevertheless, the rate constants are lower than in the case of resveratrol and (-)-epicatechin, though higher than in the case of vanicoside B (Table 3). Similar results were reported by Bruck et al. for mitoxantrone (also belonging to anthraquinones family), for which the rate constants were below the values determined for (-)-epicatechin and resveratrol [38].

Referring to the results mentioned in Section 3.2, stopped-flow experiments revealed that water residue fraction contains a certain concentration of compounds reactive with LPO Compound I and Compound II. However, registered observed rate constants were the lowest among tested fractions, disqualifying them among the candidates for LPO activators.

As shown in 3.2, all four fractions that exhibited properties promoting the synthesis of OSCN^–^ ions showed the opposite effect at higher concentrations, leading to a complete inhibition with sufficiently high concentrations. In the light of results obtained using the stopped-flow technique, a possible explanation for this phenomenon may be the competition regarding the access to Compound I between SCN^–^ ions and organic substrates, which gets fiercer with the increasing reaction rate constant for the reaction of organic substrate with Compound I.

### 3.4. Assessment of the Ability to Increase the Antimicrobial Activity of the LPO System

**Figure 6:**
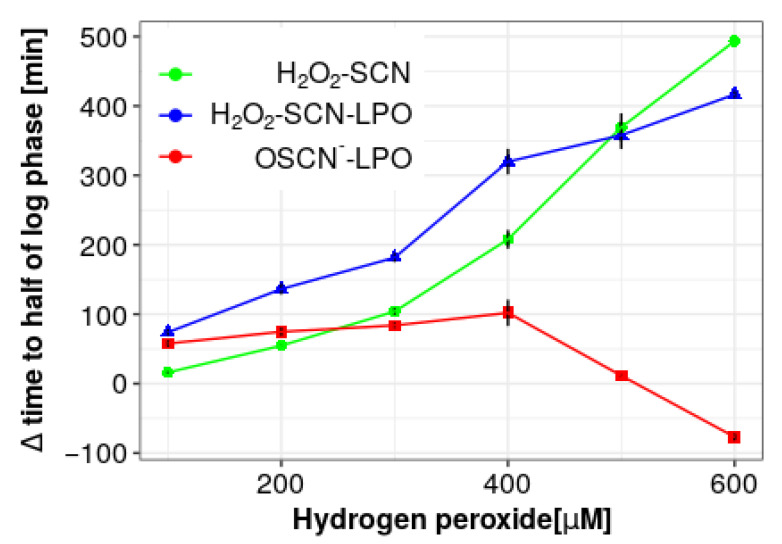
Green line with dots represents concentration-dependent toxicity against *S. mutans* by lactoperoxidase substrates (H_2_O_2_ and SCN^–^ ions) and is expressed as time difference to reach half of log phase between the sample containing substrates and sample containing *S. mutans* only. Similarly, blue line with triangles represents the toxicity dependent on substrates and products of lactoperoxidase system generated in situ. Red line with squares is the difference between the whole LPO system toxicity and substrates-only toxicity, which corresponds to the toxicity dependent on the LPO products. Positive value of that parameter indicates an antimicrobial effect of the system, while negative value indicates protection against the toxicity of the substrates.

To evaluate the ability to enhance the antimicrobial properties of the LPO system, extracts fractions and individual polyphenols that showed the ability to increase the rate of OSCN^–^ ion synthesis were used. These included: (-)-epicatechin, resveratrol, ether fractions of all species, and the DCM fraction of *R. x bohemica* (see Section 3.2). In the developed model, thiocyanate ions and lactoperoxidase were used at the concentration levels present in human physiological saliva (3 mM and 50 mM, respectively). Hydrogen peroxide was used in a concentration of 300 µM, which is several times higher than the physiological one [3]. The optimal (H_2_O_2_) was selected experimentally to achieve the highest possible antimicrobial effect dependent solely on the OSCN^–^ ion, with a simultaneous miniscule antimicrobial effect (tuned to be as small as possible) induced by H_2_O_2_ and SCN^–^ without the presence of LPO, as shown in Figure 6. With increasing the hydrogen peroxide concentration up to 400 µM, an enhancement of the antimicrobial effect, depending on the LPO system activity, was observed. At concentrations above 400 µM, the toxic effect, generated solely by the presence of hydrogen peroxide, became more significant than that of the LPO system antimicrobial product. In that situation, (pseudo)halogenation activity of LPO, in which hydrogen peroxide is consumed, paradoxically causes LPO to exhibit protective activity against hydrogen peroxide toxicity.

**Figure 7:**
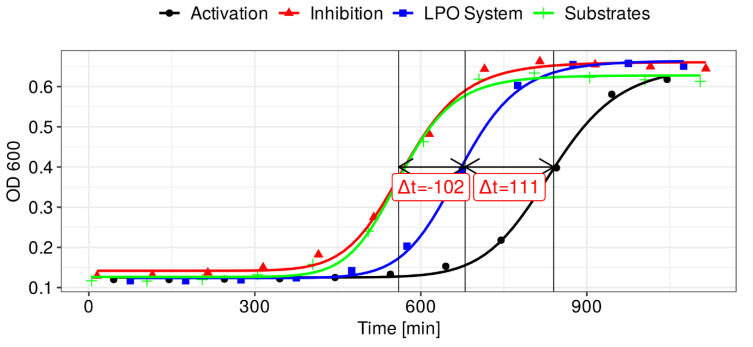
Examples of growth curves recorded spectrophotometrically at 600 nm. The green line shows the growth of *S. mutans* in the presence of substrates of the LPO system (H_2_O_2_ and SCN^–^ ions). The blue line shows the growth in the presence of the complete LPO system (H_2_O_2_–SCN–LPO). The black line shows microbial growth in the presence of the complete LPO system and 12 µM (-)-epicatechin. Vertical lines indicate the time taken by the sample containing the activated LPO system and the LPO system to reach half the logarithmic growth phase in the graph. The red line shows microbial growth in the presence of the complete LPO system and 760 µM (-)-epicatechin.

The inhibitory effect on the microbial growth induced by the LPO system was manifested in a delay of the start of the logarithmic growth phase, as shown in Figure 7. At the same time, no statistically significant increase in the duration of the logarithmic growth phase was observed, and no significant differences were found between the optical density of the cultures by the end of the logarithmic growth phase when compared with reference samples. Bacterial growth entering a logarithmic phase, even in the samples where the LPO system was activated, may be associated with the gradual depletion of hydrogen peroxide, as this substrate was added only at the beginning of the experiment. Due to this, the bacteriostatic effect of the LPO system remained only for a limited time.

**Figure 8:**
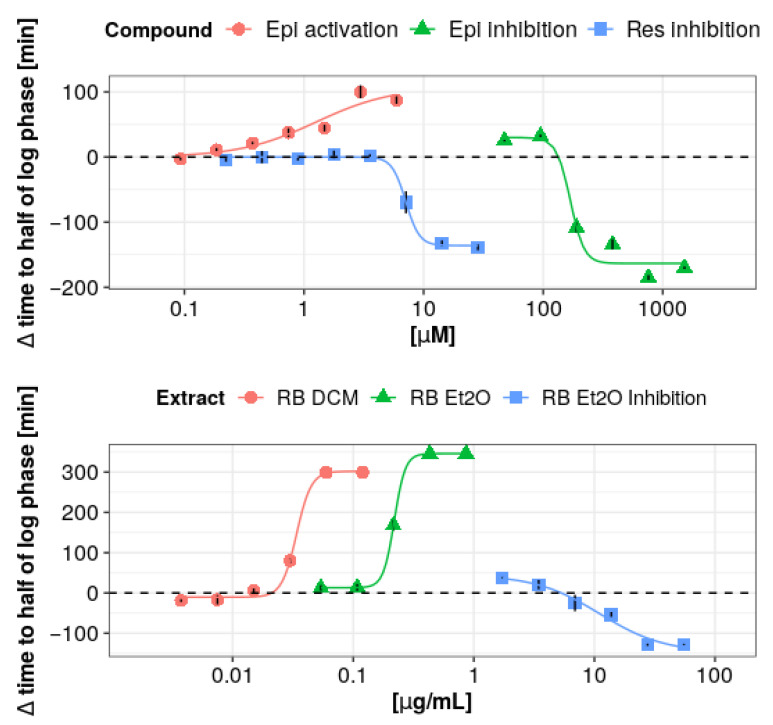
Dose–response curves (the Hill model) for the analyzed polyphenols and extracts that showed activating or inhibiting antimicrobial effect on the LPO system. Abbreviations: Epi, (-)-epicatechin; Res, resveratrol; RB, *R. x bohemica*; DCM, dichloromethane; Et2O, diethyl ether.

In order to evaluate the activating/inhibitory effect of selected individual polyphenols and extract fractions, the Δ*t* parameter was determined (Table 4), viz. the difference between the time needed to reach the half of the logarithmic growth phase by the sample containing full LPO system and by the sample containing full LPO system supplemented with the individual polyphenol or extract fraction. Δ*t* values were positive when the antimicrobial activity of the LPO system was enhanced, whereas the opposite effect was manifested by negative values of this parameter. The mentioned parameter was determined for a series of 2-fold dilutions of each tested fraction/compound. For evaluation of activating/inhibitory potency of selected individual polyphenols and extract fractions, obtained Δ*t* was plotted against the concentration of the sample. Next, the obtained dependence was fitted using the Hill method to obtain dose–response curves (Figure 8). This approach allowed calculation of the EC_10/50/90_ in the case of the activating effect and IC_10/50/90_ in the case of the inhibiting effect (Table 4).

In the case of (-)-epicatechin, the nature of the effect depends on the concentration. Similarly, as in the analysis of the kinetics of OSCN^–^ ion synthesis at lower (-)-epicatechin concentrations, the activation effect was exhibited (extension of the time needed to reach half the logarithmic growth phase of the sample containing the LPO system with (-)-epicatechin compared with the sample containing the LPO system alone-Table 4), whereas an inhibitory effect was observed at higher concentrations (suppression of the growth-inhibitory effect). This can be explained by the competition between (-)-epicatechin and SCN^–^ ions to access Compound I, which is supported by the relatively high rate constants for the epicatechin oxidation by Compound I. At high concentrations of (-)-epicatechin, the peroxidation cycle reaction is favored, leading to the attenuation of the (pseudo)halogenation cycle or complete inhibition above a certain threshold. This, in turn, leads to the impairment of the antimicrobial effect of the LPO system. What is important is that both EC_50_ and IC_50_ in the microbiological model attain greater values than in the kinetic model with Ellman’s reagent.

In contrast to the kinetic studies with Ellman’s reagent, in the case of resveratrol, only the inhibition of LPO system antimicrobial properties (IC_50_ 7.07 vs. 0.01 µM in kinetic model) was registered, and no activating effect was observed. Lack of that effect may be related to a 4-fold lower activation percentage (Table 1) of this compound compared with (-)-epicatechin, thus resulting in weaker activation of the enzyme, which does not translate into a noticeable biological effect.

The diethyl ether fraction of *R. x bohemica* extract, similar to the kinetic assay with Ellman’s reagent, showed an activating effect at lower concentrations and an inhibitory effect at higher concentrations. In the case of the DCM fraction of *R. x bohemica* extract, only an activating effect with regard to the antimicrobial properties of the LPO system was observed. Nevertheless, it should be noted that this fraction was characterized by such strong antimicrobial properties that when tested alone it exhibited an inhibitory effect on the growth of *S. mutans*. This deemed the study of the effect of this fraction on the LPO system in a microbiological model at a concentration above 2 µg/mL not possible in a sufficiently wide range of concentrations.

It should be noted that the analyzed fractions displayed an approximately 3 times stronger activation than (-)-epicatechin (**Δ*t*_max_**: RB DCM = 299 min, RB Et2O = 345 min vs (-)-epicatechin 111 min). These results do not match with the results obtained in the kinetic model with Ellman’s reagent, wherein the activating potential of these fractions in the biological model is surprisingly high in relation to (-)-epicatechin, which in the kinetic model was selected as the most promising substance. This indicates that the final biological effect of the modulated lactoperoxidase system is influenced by environmental factors such as changes in environment pH, bacterial production, and decomposition of hydrogen peroxide, and finally potential synergistic phenomena between LPO system modulation and direct action against microorganisms.

### 3.5. Antimicrobial Activity of Extracts from Reynoutria sp.

The MIC and MBC values for the tested extracts and their fractions are given in Table 5. Among acetone extracts, *R. japonica* extract (MIC 0.15 mg/mL) showed the strongest inhibitory effect on the growth of microorganisms. The DCM fraction showed the strongest inhibitory effect as well, and in the tested concentration range, no antimicrobial activity of this fraction was observed (MBC > 1.2). The strongest bactericidal activity was recorded for the ethyl acetate fraction (MBC 0.60 mg/mL for each species).

Smullen et al. showed that the antimicrobial properties of plant extracts against *S. mutans* increased with the content of polyphenols [39]. This dependence is also noticeable in the results of our research. The water fraction with about 20 times lower content of polyphenols [29] than the ethyl acetate fraction showed no inhibitory properties for the growth of *S. mutans* in the studied concentration range in the case of *R.* x *bohemica* and *R. sachalinensis* and very weak inhibitory properties *for R. japonica*. On the other hand, butanol, diethyl ether, and ethyl acetate fractions rich in polyphenols were characterized by strong inhibitory properties toward *S. mutans*. Interestingly, the DCM fraction of *R.* x *bohemica* characterized by a relatively low polyphenol content [29] showed the strongest inhibitory effect toward *S. mutans* among the tested fractions, with no bactericidal properties in the studied concentration range. This effect can be associated with the presence of high concentrations of emodin and anthraquinone derivatives in this fraction, which in the studies by Xiang et al. had anti-biofilm activity related not to direct bactericidal activity but to the impaired adhesion and proliferation in the early stages of biofilm formation [40].

### 3.6. Physiological Relevance

Dental caries is a process of an extrinsic origin that leads to the demineralization and proteolysis of hard dental tissues [41,42]. It is associated with the production of organic acids by sugar-fermenting bacteria, using mainly simple sugars [12]. Nowadays, caries prophylaxis is mainly based on fluoride additions to oral hygiene products [43], oral rinses with chemotherapeutics (i.e., chlorhexidine) [44], and in the most severe cases, antibiotics. Despite access to prophylaxis, dental caries is a serious health problem on a global scale; therefore, there is an emphasis on searching for new therapies, particularly based on natural products [45,46]. The use of *Reynoutria* sp. extracts in dental caries prevention may be beneficial for two reasons. Firstly, phenolic compounds found in large amounts in these extracts can be a potential source of host LPO system activator, leading to the increase of innate immunity defense mechanisms. Secondly, *Reynoutria* sp. extracts are able to directly affect cariogenic bacteria growth, viability, metabolism, and biofilm formation. Although, *Reynoutria* sp. healing properties in oral cavity diseases was known in traditional medicine for centuries [28], this plant has aroused increasing interest in recent years due to a growing demand for new therapies for bacterial diseases, including the treatment and prevention of dental caries.

## 4. Conclusions

The reported studies prove the activating effect of peroxidation cycle constituents on the antimicrobial properties of the LPO system in the microbiological model. Our research revealed that phenolic compounds contained in the rhizomes of three tested species of *Reynoutria* have the ability to modulate the activity of the lactoperoxidase system. Importantly, the obtained extracts contained compounds leading to both the activation and inhibition of the (pseudo)halogenation cycle of LPO, responsible for the antimicrobial properties of this enzyme. The activation properties were mainly due to low-molecular-weight phenolic compounds, such as stilbene derivatives and flavan-3-ols, while the inhibitory effect should be attributed to strongly cross-linked polyphenols, such as proanthocyanidins, lignin oligomers, or macromolecular phenylpropanoid sucrose derivatives. Among tested species, *R.* x *bohemica* dichloromethane and diethyl ether fractions showed the most promising effect associated with significant activation of LPO antimicrobial action. In the case of polyphenols, only (-)-epicatechin exhibited activation properties in the microbiological model.

The results of our research show that the effectiveness of pharmaceutical preparations based on the extracts from *Reynoutria* sp. in the hygiene and treatment of oral diseases is based not only on the anti-inflammatory properties and direct antimicrobial effect of these extracts but also on the modulation of the LPO system. The obtained results may also indicate that extracts from other plants used as components of oral hygiene preparations and whose activity so far has been associated only with a direct effect on microorganisms may also have an activating effect on the LPO system, increasing their effectiveness in the prevention of caries. Nevertheless, further studies are required to take into account the possible phytochemical variability of the plant, to develop an extraction process that allows to obtain fractions with the smallest amount of inhibitory compounds, and to extend the research to multispecies commensal–opportunistic microbiological models that better reflect the biochemical and ecological conditions prevailing in the dental plaque.

## Figures and Tables

**Table 1:** Half-maximal inhibitory concentration (IC_50_), half-maximal effective concentration (EC_50_), and the percentage of activation of individual fractions obtained for extracts originating from the rhizomes of *Reynoutria* sp. Species, extracts fractions at selected solvents, and analyzed polyphenols. The means were calculated from 4–8 replicates; after ‘±’, a standard error is given. Numbers/letters in superscript indicate significant differences between extracts/fractions/compounds with a given number obtained with ANOVA with post-hoc Tukey test.

Extract or Fraction	Species	No	IC_50_ (µg/mL)	EC_50_ (µg/mL)	Activation (%)
Acetone	*R. x bohemica*	1	3.203 ± 0.334 ^10,11,12,13^	-	-
	*R. japonica*	2	2.806 ± 0.253 ^10,11,12,13^	-	-
	*R. sachalinensis*	3	2.657 ± 0.416 ^10,11,12^	-	-
DCM	*R. x bohemica*	4	2.525 ± 0.176 ^7,8,9,16,17,18^	0.034 ± 0.001 ^7,8,9^	18.71
	*R. japonica*	5	3.266 ± 0.226 ^6,10,11^	-	-
	*R. sachalinensis*	6	5.486 ± 0.759 ^7,8,9,17^	-	-
Diethyl eter	*R. x bohemica*	7	1.049 ± 0.044 ^4,6,9,10,11,13^	0.009 ± 0.002 ^4^	15.56
	*R. japonica*	8	0.794 ± 0.067 ^4,6,9,10,11,12,13^	0.006 ± 0.001 ^4^	25.51
	*R. sachalinensis*	9	1.155 ± 0.059 ^4,6,7,8,10,11,12,13,14,15,18^	0.008 ± 0.003 ^4^	10.69
Ethyl acetate	*R. x bohemica*	10	1.199 ± 0.100 ^1,2,3,5,7,8,9,16,17,18^	-	-
	*R. japonica*	11	1.771 ± 0.096 ^1,2,3,5,7,8,9,16,17,18^	-	-
	*R. sachalinensis*	12	1.904 ± 0.240 ^8,9,16,17,18^	-	-
Butanol	*R. x bohemica*	13	2.663 ± 0.316 ^1,2,7,8,9,16,17,18^	-	-
	*R. japonica*	14	2.267 ± 0.315 ^9,17^	-	-
	*R. sachalinensis*	15	1.056 ± 0.169 ^9,17^	-	-
Water	*R. x bohemica*	16	3.062 ± 0.674 ^4,10,11,12,13^	-	-
	*R. japonica*	17	10.039 ± 3.659 ^4,6,10,11,12,13,14,15^	-	-
	*R. sachalinensis*	18	2.240 ± 0.316 ^4,9,10,11,12,13^	-	-
**Compound**			**IC_50_ (µM)**	**EC_50_ (µM)**	**Activation (%)**
(-)-Epicatechin		a	74.081 ± 3.624 ^b,d^	1.314 ± 0.239 ^b^	46.31
Resveratrol		b	0.423 ± 0.018 ^a,d^	0.009 ± 0.002 ^a^	12.58
Emodin		c	-	-	-
Vanicoside B		d	1.000 ± 0.074 ^a,b^	-	-

**Table 2:** The observed rate constants for both investigated reactions in the presence of analyzed extracts in concentration of 5 µg/mL at 15 °C. The means were calculated from 4–8 replicates, after ‘±’, a standard error is given. For reactions where the kinetic curve was double-exponential, the value of the lower observed rate constant is given as k_obs2_. Numbers in superscript indicate significant differences between extracts/fractions of given *Reynoutria* species with a given number obtained with ANOVA with post-hoc Tukey test.

Species	Solvent	No	k_obs1_ (s^−1^)	k_obs2_ (s^−1^)	k_obs1_ (s^−1^)	k_obs2_ (s^−1^)
			Compound I -> Compound II		Compound II -> Native LPO	
*R.* x *bohemica*	acetone	1	18.71 ± 4.15 ^2,3,4,6^	-	2.35 ± 0.30 ^2,3,5,6^	-
	DCM	2	87.36 ± 11.82 ^1,3,5,6^	3.36 ± 0.50	18.72 ± 6.23 ^1,3,5,6^	0.85 ± 0.18 ^3,4,6^
	Et_2_O	3	485.60 ± 19.86 ^1,2,4,5,6^	-	97.65 ± 11.14 ^1,2,4,5,6^	6.24 ± 1.08 ^2,4,5,6^
	EtAc	4	98.87 ± 20.85 ^1,3,5,6^	-	14.57 ± 1.22 ^3,5,6^	3.86 ± 0.88 ^2,3,5,6^
	butanol	5	15.64 ± 2.80 ^2,3,4,6^	2.62 ± 0.62	0.38 ± 0.07 ^1,2,3,4^	0.10 ± 0.04 ^3,4^
	Water	6	2.01 ± 0.22 ^1,2,3,4,5^	-	0.23 ± 0.08 ^1,2,3,4^	0.01 ± 0.01 ^2,3,4^
*R. japonica*	acetone	7	55.38 ± 2.31 ^8,11,12^	-	5.81 ± 0.87 ^8,9,10,11,12^	-
	DCM	8	130.90 ± 26.22 ^7,11,12^	2.63 ± 0.52	28.76 ± 6.71 ^7,9,10,11,12^	3.43 ± 1.13 ^11,12^
	Et_2_O	9	98.87 ± 20.85 ^11,12^	-	14.57 ± 1.22 ^7,8,11,12^	3.86 ± 0.88 ^11,12^
	EtAc	10	79.04 ± 13.57 ^11,12^	-	14.29 ± 0.61 ^7,8,11,12^	4.20 ± 0.87 ^11,12^
	butanol	11	20.03 ± 2.56 ^7,8,9,10,12^	2.97 ± 0.54	0.66 ± 0.16 ^7,8,9,10^	0.27 ± 0.10^8,9,10^
	Water	12	2.11 ± 0.35 ^7,8,9,10,11^	-	0.33 ± 0.05 ^7,8,9,10^	0.02 ± 0.01 ^8,9,10^
*R. sachalinensis*	acetone	13	33.55 ± 8.40 ^15,17,18^	-	0.84 ± 0.22 ^15,16^	-
	DCM	14	16.21 ± 2.21 ^15,16,18^	2.11 ± 0.21	1.46 ± 1.17 ^15^	0.41 ± 0.22 ^15,18^
	Et_2_O	15	156.43 ± 16.57 ^13,14,16,17,18^	-	22.38 ± 1.73 ^13,14,16,17,18^	8.46 ± 1.79^14,16,17,18^
	EtAc	16	48.62 ± 5.06 ^14,15,17,18^	-	4.18 ± 0.79 ^13,15,17,18^	1.52 ± 0.31 ^15,18^
	butanol	17	19.70 ± 3.90 ^13,15,16,18^	3.37 ± 0.88	0.40 ± 0.07 ^15,16^	0.11 ± 0.07 ^15^
	Water	18	1.77 ± 0.21 ^13,14,15,16,17^	-	0.42 ± 0.03 ^15,16^	0.06 ± 0.01 ^14,15,16^

**Table 3:** Apparent second-order rate constants for the reaction of selected extracts and polyphenols with LPO Compounds I–II.

**Acetone Extract**	**k (ml µg^−1^ s^−1^)** **Compound I -> Compound II**	**k (ml µg^−1^ s^−1^)** **Compound II -> Native LPO**
*R. sachalinensis*	1.07 ± 0.10	0.07 ± 0.01
*R. japonica*	13.18 ± 0.31	1.38 ± 0.10
*R. x bohemica*	12.44 ± 0.55	0.49 ± 0.03
**Substrate**	**k (M^−1^ s^−1^)** **Compound I -> Compound II**	**k (M^−1^ s^−1^)** **Compound II -> Native LPO**
(-)-Epicatechin	(4.3 ± 0.3) × 10^6^	(2.6 ± 0,4) × 10^6^
Resveratrol	N/D *	(1.71 ± 0.1) × 10^7^ **
Emodin	N/D ***	(2.5± 0.2) × 10^5^
Vanicoside B	(1.9 ± 0.3) × 10^6^	(3.6 ± 0.5) × 10^3^

* The reaction rates were too fast to be followed by stopped-flow technique. ** Measured at 5 °C temperature to slow down the reaction. *** The kinetic traces run did not converge the exponential character.

**Table 4:** ED_10/50/90_ values determined for compounds and extracts activating the antimicrobial properties of the LPO system and IC_10/50/90_ in the case of compounds/extracts inhibiting the antimicrobial activity. The ∆*t_max_* parameter determines the maximum recorded effect strength.

**Compound**	**Effect Type**	**ED10/IC10 (µM)**	**ED50/IC50 (µM)**	**ED90/IC90 (µM)**	**Δ*t*_max_ (min)**
(-)-Epicatechin	Activation	0.13 ± 0.33	2.07 ± 1.01	14.57 ± 4.04	111 ± 11
(-)-Epicatechin	Inhibition	106.96 ± 9.58	161.106 ± 16.14	494.29 ± 168.37	−185 ± 4
Resveratrol	Inhibition	5.17 ± 2.37	7.07 ± 0.27	9.67 ± 4.44	−138 ± 4
**Extract**	**Effect Type**	**ED10/IC10 (µg/mL)**	**ED50/IC50 (µg/mL)**	**ED90/IC90 (µg/mL)**	**Δ*t*_max_ (min)**
*R. x bohemica* (DCM)	Activation	0.02 ± 0.01	0.03 ± 0.01	0.05 ± 0.01	299 ± 7
*R*. *x bohemica* (diethyl ether)	Activation	0.17 ± 0.10	0.22 ± 0.01	0.28 ± 0.19	345 ± 13
*R. x bohemica* (diethyl ether)	Inhibition	12.40 ± 7.72	15.02 ± 7.42	18.17 ± 7.81	−128 ± 3
*R. japonica* (diethyl ether)	no effect	-	-	-	-
*R. sachalinensis* (diethyl ether)	no effect	-	-	-	-

**Table 5:** MIC and MBC values for all analyzed extracts.

Fraction	Species	MIC (mg/mL)	MBC (mg/mL)
Acetone	*R. x bohemica*	0.30	0.60
	*R. japonica*	0.15	0.60
	*R. sachalinensis*	0.30	1.20
DCM	*R. x bohemica*	0.08	>1.20
	*R. japonica*	0.30	>1.20
	*R. sachalinensis*	0.15	>1.20
Etyl acetate	*R. x bohemica*	0.30	0.60
	*R. japonica*	0.15	0.60
	*R. sachalinensis*	0.30	0.60
Diethyl eter	*R. x bohemica*	0.30	>1.20
	*R. japonica*	0.15	>1.20
	*R. sachalinensis*	0.60	>1.20
Butanol	*R. x bohemica*	1.20	>1.20
	*R. japonica*	0.30	1.20
	*R. sachalinensis*	0.30	0.60
Water	*R. x bohemica*	>1.20	>1.20
	*R. japonica*	1.20	>1.20
	*R. sachalinensis*	>1.20	>1.20

## Data Availability

Not applicable.
